# Association of childhood maltreatment with internalising and externalising disorders in trauma-exposed adolescents

**DOI:** 10.4102/sajpsychiatry.v24i0.1266

**Published:** 2018-09-20

**Authors:** Leigh L. van den Heuvel, Milo Koning, Jani Nöthling, Soraya Seedat

**Affiliations:** 1 Department of Psychiatry, Faculty of Medicine and Health Sciences, Stellenbosch University, South Africa

## Abstract

**Introduction:**

South African adolescents experience high levels of trauma, including various types of childhood maltreatment. Different types of maltreatment often co-occur. Previous research suggests that childhood maltreatment provokes a latent liability to internalising and externalising dimensions of psychopathology. Our objective was to examine the effects of childhood maltreatment on internalising and externalising disorders in trauma-exposed adolescents and to assess the mediating effect of post-traumatic stress disorder (PTSD) on these associations.

**Methods:**

A cross-sectional study was conducted with 262 trauma exposed adolescents (aged 12–18 years) in South Africa. Childhood maltreatment and PTSD severity were assessed using the Childhood Trauma Questionnaire and the Child PTSD Checklist, respectively. Psychiatric disorders were diagnosed utilising the Kiddie Schedule for Affective Disorders and Schizophrenia – Present and Lifetime version – and were grouped into internalising and externalising disorders. Hierarchal logistic regression was used to assess the association between childhood maltreatment types and internalising and externalising disorders, controlling for statistically significant socio-demographic characteristics, with PTSD severity added to the final model as a potential mediator.

**Results:**

Sexual abuse was significantly associated with internalising disorders (*B* = 0.07, *p* = 0.011), although this effect was mediated by PTSD severity (*B* = 0.05, *p* = 0.001; not included as an internalising disorder). In contrast, physical abuse (*B* = 0.09, *p* = 0.004) and gender (*B* = 0.70, *p* = 0.035) were associated with externalising disorders, but the addition of PTSD severity did not significantly alter these associations.

**Conclusion:**

The association between sexual abuse and internalising disorders was fully mediated by PTSD symptom severity. Gender and physical abuse severity, but not PTSD severity, was associated with the presence of externalising disorders. Adolescents displaying internalising or externalising psychopathology need to be assessed for exposure to childhood physical and sexual abuse and PTSD comorbidity.

